# Perspectives of EGFR co-targeting with nanocarriers for anti-TNBC purposes

**DOI:** 10.1016/j.gendis.2025.102026

**Published:** 2026-01-03

**Authors:** Piotr Białecki, Miłosz Adamiak, Elżbieta Pędziwiatr-Werbicka, Agnieszka Robaszkiewicz

**Affiliations:** a Department of General Biophysics, Faculty of Biology and Environmental Protection, University of Lodz, Pomorska 141/143, Lodz 90-236, Poland; b Bio-Med-Chem Doctoral School of the University of Lodz and Lodz Institutes of the Polish Academy of Sciences, University of Lodz, Banacha 12/16, Lodz 90-237, Poland

**Keywords:** Cancer therapy, EGFR, Liposome, Nanoparticle, TNBC

## Abstract

Triple-negative breast cancer (TNBC) is a particularly aggressive and therapeutically challenging subtype of breast tumor, frequently marked by the relatively high expression of epidermal growth factor receptor (EGFR). As a tyrosine kinase, this enzyme plays a crucial role in driving tumor cell proliferation, progression, and metastases. This review describes the restricted clinical benefits of single EGFR targeting, its expression profile across TNBC subtypes, and the genomic and non-genomic determinants of *EGFR* transcription, followed by the possibilities of combinatorial and multi-targeted therapy approaches. By analyzing patterns of gene expression in *EGFR*-high and *EGFR*-low samples, additional co-targeting factors were identified, which could enhance the efficacy of anti-EGFR strategies in TNBC and serve as more precise and personalized treatment standards. The impact of simultaneous EGFR and selected factors on patient survival is presented. Finally, we mention the available nanocarriers capable of delivering two inhibitors, siRNAs, and antibodies, or their mixtures, for the reduction of EGFR and co-expressed partners.

## Introduction

### Breast cancer types and short characteristics of TNBC

Breast cancer is the most common cancer among women, representing approximately 30% of all cancer diagnoses and contributing to 14% of cancer-related deaths.^1^ These statistics highlight the urgency of advancing prevention, diagnostic approaches, and therapeutic interventions.

Breast cancer is typically classified by a specific receptor expression within the cell nucleus, including hormone receptors such as progesterone (PR), estrogen (ER), and the human epidermal growth factor receptor 2 (HER2). The profile of their abundance or deficiency plays a critical role in distinguishing tumor subtypes, with four primary breast cancer types identified. Luminal subtypes A and B exhibit strong steroid receptor expression (progesterone and estrogen) that influences tumor behavior and response to treatment.^2^ HER2-enriched is characterized by frequent amplification of HER2 (80%).^3^ The last type, the triple-negative breast cancer (TNBC), is further divided into six subgroups: androgen receptor luminal (LAR), mesenchymal (M), basal-like 1/2 (BL1 and BL2), immunomodulatory (IM), and mesenchymal stem-like (MSL). The subdivisions derive from the variability in transcriptomes of breast cancer cells and their interaction with immune cells, which is particularly strong in the IM subtype. BL1 and BL2 are characterized by a higher expression of cell cycle and DNA damage response genes. The LAR subtype is frequently found in patients with decreased relapse-free survival and is featured by the androgen receptor. The MSL and M subtypes share the enrichment of genes, which contribute to similar biological processes such as cell motility, cellular differentiation, and growth pathways.^4^

### TNBC treatment approaches and outcomes

The most common anti-cancer treatment for receptor-positive breast cancers comprises hormonal or anti-HER2 approaches. Hormone or endocrine therapy is often used for tumors characterized by the presence of estrogen or progesterone receptors and aims to compete with hormones, thereby blocking their downstream signaling. Although HER2 belongs to the family of epidermal growth factor receptor (EGFR) tyrosine receptor kinases, it does not require interaction with specific ligands, but is activated by forming homo- or heterodimers with other family members, also with EGFR. Co-amplification of *HER2* and *EGFR* in breast cancer patients was associated with a higher possibility of distant metastasis and lower overall and disease-free survival.^5^ Anti-HER2 therapies target HER2 on the cell surface with monoclonal antibodies and antibody-drug conjugates, whereas HER2 activity is inhibited with tyrosine kinase inhibitors to limit the proliferation and invasion of cancer cells.^6^ However, high EGFR expression mediates resistance to the HER2 antibody drug conjugate,^7^ therefore combining HER2 and EGFR therapies that combine an EGFR inhibitor with a HER2-targeted approach or dual EGFR/HER2 inhibitors are being proposed to treat EGFR-HER2 positive cancers.^8^

The above-mentioned approaches are not applicable to TNBC, which accounts for approximately 9%–16% of all breast cancers,^9^ and lacks estrogen, progesterone, and HER2 receptors, whereas EGFR level varies among TNBC subtypes. Removal of the tumor is often insufficient, as TNBC is characterized by a high metastatic rate in other tissues and exhibits a high recurrence, with currently the worst prognosis among all breast cancer subtypes.^10^ The majority of TNBCs (95%) are classified histologically as invasive mammary carcinomas of no specific type (or invasive ductal carcinomas) and with no distinctive histological characteristics.^11^ Patients diagnosed with TNBC have an overall poor prognosis, and according to O’Reilly et al,^12^ 25% will develop recurrent disease with distant metastases. For patients with advanced or stage IV disease, the median overall survival is approximately 12 months, and less than 20% of patients survive four years.

Although TNBC represents the most aggressive breast cancer subtype, it lacks targeted therapies; hence, therapeutic approaches rely mostly on radiotherapy, adjuvant or neoadjuvant chemotherapy, and immune anti-PD-L1 therapies, the first two being often associated with significant adverse effects. The treatment options for drug-resistant phenotypes are mostly restricted to two PARP inhibitors, olaparib (Lynparza) and talazoparib (Talzenna). The treatment statistics for the pathological complete response rate with paclitaxel-olaparib are 55.1% versus paclitaxel-carboplatin at 48.6%^13^ in *BRCA1/2* mutated cases, and antibody-drug conjugate sacituzumab govitecan (Trodelvy) for Trop-2-positive cancers. However, the statistics for PARP inhibitors are not optimistic. The survival rate with talazoparib drops from 71% to 42% and 27% after 12, 24, and 36 months of treatment, respectively.^14^ The substantial benefit of olaparib over carboplatin has not been reported in paclitaxel-based combinatorial approaches based on the pathological complete response, indicating that no tumor cells were present in the primary tumor site or lymph nodes after neoadjuvant treatment.^13^ Platinum-based neoadjuvant chemotherapy significantly increased the pathological complete response rate from 37.0% to 52.1%.^15^ Therefore, the search for the molecular hallmarks of TNBC subtypes and their identification is crucial for extending the small list of treatment approaches, particularly in advanced tumors, to improve treatment efficacy. High molecular heterogeneity raises a significant challenge in the identification of a unified mechanism of chemoresistance and for the development of effective therapeutic strategies.^1^

### EGFR is in the spotlight, with restricted clinically proven activity as a target

Observations that signaling cascades downstream of EGFR promote tumor growth and survival prompted the development of anti-EGFR approaches, leading to clinical trials. Putri et al^16^ suggested that EGFR expression may predict patient life expectancy, claiming that any higher levels of expression had a risk of lower survival. Approximately 50% of the cases of TNBC and inflammatory breast cancer overexpress EGFR, with this protein emerging as a potential marker and target for anti-cancer therapies in EGFR-expressing TNBC. EGFR-directed treatments typically involve two categories of agents: monoclonal antibodies targeting the extracellular domain of EGFR, such as cetuximab, panitumumab, and matuzumab, and small-molecule tyrosine kinase inhibitors targeting the catalytic domain of the EGFR receptor that include gefitinib and erlotinib.^17^ Inhibition of EGFR signaling enhances the chemosensitivity of TNBC cells by altering the apoptotic signaling networks. However, after two decades of research, there is no convincing evidence for the clinical efficacy of anti-EGFR treatment in the cohort of TNBC or on other tumor types.^18^

Chimeric antigen receptor engineered T (CAR-T) cells have also been used as potential cancer therapies, with the analysis of EGFR CAR-T therapy focused on breast cancer lines MDA-MB-231, MDA-MB-468, MCF-7^18^, and T47D.^19^ Some success in CAR-T-based therapy that targets EGFR-expressing tumors has been reported in a mouse preclinical model. Although treatment with CAR-T cells has produced remarkable clinical responses against certain subsets of B cell leukemia or lymphoma, there are many limitations to the therapeutic efficacy of CAR-T cells in solid tumors, in particular, the expression of most tumor-associated antigens at lower levels in vital organs, which results in on-target/off-tumor toxicity.^20^ Additionally, this method has been associated with the possibility of an immune system reaction (inflammation) and the release of large amounts of cytokines. Neurotoxicity or immune effector cell-associated neurotoxicity syndrome may also occur.^19^ The emergence of such reactions necessitates the use of other techniques based on the use of small interfering RNAs (siRNAs) or inhibitors.

In this paper, the current views on anti-cancer therapies targeting EGFR in TNBC and the possible limitations of former anti-EGFR approaches were revised. Using multidimensional cancer genomic and transcriptomics datasets, as well as bioinformatic tools such as cBioPortal and Xena Browser, the expression profile and copy number alterations of EGFR were shown, defining EGFR abundance in various breast cancer subtypes. Additionally, the EGRF accompanying markers in TNBC were examined. These can be utilized for dual combinatorial inhibition or silencing with EGFR to improve the efficacy of anti-EGFR approaches. Finally, the most expedient nanocarriers were reviewed. Nanocarriers may assist in the successful delivery of two water-soluble inhibitors or, in particular, siRNAs, directly to tumor cells.

### EGFR as a target for intervention in TNBCs

#### *EGFR signaling*

**Figure 1:**
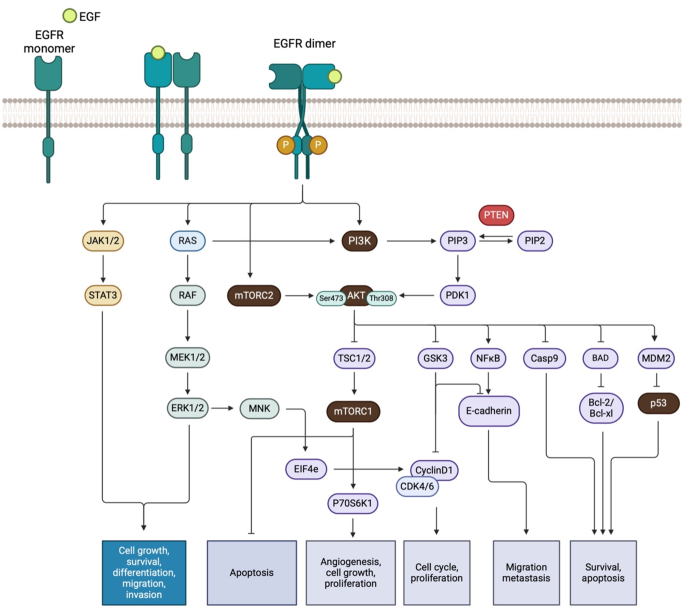
Schematic presentation of signalling pathways downstream of EGFR and the processes they regulate (based on the studies by Fisher et al^18^ and Xia et al^19^). JAK1/2, Janus-activated kinase 1/2; RAS, Rat sarcoma virus; PI3K, phosphoinositide 3-kinases; PIP3, phosphatidylinositol (3,4,5)-trisphosphate; PIP2, phosphatidylinositol 4,5-bisphosphate; PTEN, phosphatase and tensin homolog deleted on chromosome ten; STAT3, signal transducer and activator of transcription 3; mTORC1/2, mammalian target of rapamycin complex 1/2; Ser473, serine 473; Thr308, threonine 308; AKT, protein kinase B; PDK1, 3-phosphoinositide-dependent protein kinase-1; MEK1/2, mitogen-activated protein kinase 1/2; TSC1/2, tuberous sclerosis proteins 1/2; GSK3, glycogen synthase kinase 3; NFκB, nuclear factor kappa-light-chain-enhancer of activated B cells; Casp9, caspase 9; MDM2, mouse double minute 2 homolog; ERK1/2, extracellular signal-regulated kinase 1/2; MNK, MAP kinase-interacting serine/threonine-protein kinase; Bcl2, B-cell lymphoma 2; p53, tumor suppresor protein; EIF4e, eukaryotic translation initiation factor 4e; CDK4/6, cyclin dependent kinase 4/6; P70S6K1, ribosomal protein S6 kinase beta-1. The figure was created in BioRender.

EGFR (erbB-1) belongs to the erbB receptor tyrosine kinase family that also comprises HER2 (erbB-2), HER3 (erbB-3), and HER4 (erbB-4). The allosteric surface of EGFR, erbB-3, and erbB-4 contains an extracellular ligand-binding domain, a single transmembrane domain, and, in the case of EGFR, erbB-2 and erbB-4, an intracellular tyrosine kinase domain.^17^ In an unstimulated state, EGFR predominantly exists in an auto-inhibited conformation within the plasma membrane, preventing dimerization. When receptor-specific ligands such as the epidermal growth factor (EGF) and the transforming growth factor alpha (TGF-α) bind to the extracellular domain of EGFR, there is a homodimer in TNBC or heterodimers with HER2 or HER3 formed in other cell types. Dimerization initiates a series of structural rearrangements that are translocated to the cytoplasmic domain, allowing the formation of asymmetric dimers between the two juxtaposed catalytic domains and ATP-dependent phosphorylation of their tyrosine residues^21^ (Fig. 1).

#### *Role of EGFR in TNBC*

The human EGFR family includes four closely related receptors, which are transmembrane glycoproteins containing an extracellular ligand-binding domain and an intracellular receptor tyrosine kinase domain.^22^ One of the most important processes in TNBC, in which EGFR is involved, is glycolysis. As the rate of glycolysis increases, the proliferation rate of breast cancer cells increases, contributing to rapid tumor growth. EGF signalling mediates aerobic glycolysis through up-regulation of HK2 expression, accelerating the first step of glycolysis and down-regulation of PKM2 activity and slowing the last step of glycolysis.^23^ Additionally, EGFR affects the activity of the activator of transcription-3 (STAT3), contributing to the development and progression of TNBC. The EGFR monomer may activate the signal transducer activator of STAT3 in the absence of transmembrane protein TMEM25, the expression of which is frequently decreased in human TNBC. Deficiency of TMEM25 allows the EGFR monomer to phosphorylate STAT3 independent of ligand binding and thus enhances basal STAT3 activation.^24^ Another important aspect in TBNC is the low survival rate; EGFR has a role in this. The intercellular adhesion molecule-1 (ICAM-1) exhibits elevated expression levels in metastatic breast cancer and serves as a pivotal binding adaptor for EGFR activation, playing a crucial role in malignant progression. The activation of EGFR by tumor-expressed ICAM-1 initiates biased signalling within the JAK1/STAT3 pathway, consequently driving epithelial-to-mesenchymal transition and facilitating heightened metastasis without influencing tumor growth.^25^ EGFR signalling enhances aerobic glycolysis in TNBC cells, promoting tumor growth and immune escape.^23^

#### Copy number alteration and expression of EGFR in TNBC

**Figure 2:**
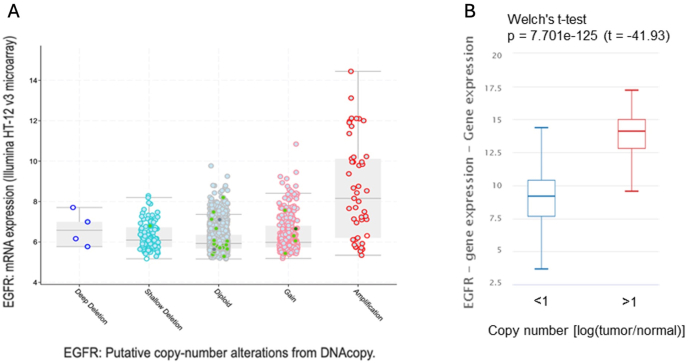
The interdependence between *EGFR* copy number alterations and mRNA level in breast cancer. **(A)** Breast cancer (METABIC, *Nature* 2012 & *Nat Commun* 2016) analyzed in cBioPortal. **(B)** Breast cancer dataset^25^ analyzed in Xena Browser.

*EGFR* is located on the short arm of human chromosome 7 and encodes a 170 kDa transmembrane protein.^26^ Genetic mutations play a significant role in altering the EGFR expression across various types of breast cancer, with gene amplification recognized as the primary type of mutation that correlates with increased mRNA level (Fig. 2A). Similarly, an elevated copy number of *EGFR* was followed by considerably higher *EGFR* transcript (Fig. 2B). However, the additional transcriptional regulation and overproduction of ligands through autocrine or paracrine mechanisms has also been described.^21^

**Figure 3:**
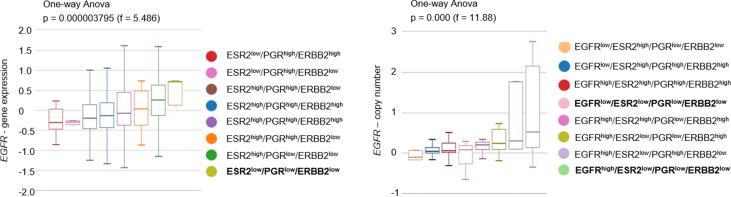
*EGFR* expression analysis. **(A, B)***EGFR* transcription (A) and copy number alteration (B) in various types of breast cancer based on the breast cancer dataset^25^ and TCGA Breast Cancer (BRACA), respectively, visualized in Xena browser.

While analyzing breast cancers that vary in the expression profile of estrogen, progesterone, and HER2 receptors, TNBC was found to be characterized by the highest mRNA level of *EGFR* (Fig. 3A), and in these cells, *EGFR* transcription was strictly linked to the *EGFR* copy number. This differed depending on the level of the three receptors: ER (*ESR2*), PR (*PGR*), and HER2 (*ERBB2*), thereby underlying the possible contribution of other, likely non-genomic factors, in defining the mRNA level of EGFR (Fig. 3B).

According to the literature, *EGFR* amplification varied among cancers and in the breast, ranging from 0.8% to 14% with reports in approximately 25% of metaplastic cases. This value is close to the frequency reported in TNBC, where it reached 24%.^27^ Tumors with elevated EGFR tend to grow more aggressively and are more likely to metastasize.^28^ Enhanced expression of EGFR in the primary tumor due to polysomy is associated with decreased survival of TNBC patients, with EGFR acting as a tumor driver.^29^ Furthermore, the increased gene copy number is associated with resistance to epidermal growth factor receptor tyrosine kinase inhibitors.^30^ Inappropriate EGFR activation in tumors is primarily attributed to gene amplification and point mutations at the gene *locus*.

**Figure 4:**
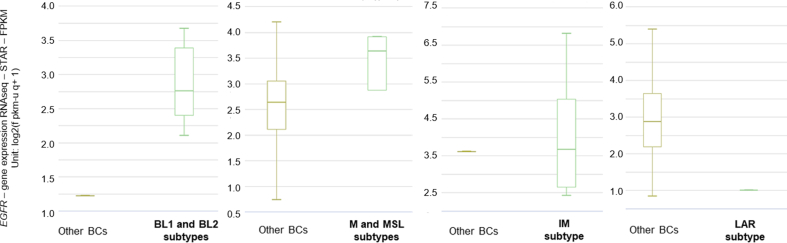
mRNA level of EGFR in TNBC subtypes. TNBC subtypes were identified based on the specific marker profile, where BL1 and BL2 are *AURKA*^*high*^*MYC*^*high*^*RAD51*^*high*^, M and MSL are *TGFB1*^*high*^*MMP2*^*high*^*CTNNB1*^*high*^, IM is *TNF*^*high*^*IL7*^*high*^*NFKB1*^*high*^*RELA*^*high*^, and LAR is *DHCR24*^*high*^*ALCAM*^*high*^*FOXA1*^*high*^*KRT18*^*high*^ based on the characteristic gene transcription profile published by Lehmann et al.^4^ Kliknij lub naciśnij tutaj, aby wprowadzić tekst. The data were taken from GDC TCGA Breast Cancer (BRCA).

Importantly, according to the GDC TCGA Breast dataset and characteristics published by Lehmann et al,^4^ all four TNBC subtypes substantially vary in EGFR yield. This finding implies that there is a need for specific subtype identification to maximize the beneficial effect of anti-*EGFR* therapies and may, at least partially, explain the lack of clinical benefits of the anti*-EGFR* approaches. In our analysis in Fig. 4, BL1/2 had the highest expression of EGFR among all considered TNBCs, but declined in the following sequence: BL1/2>M/MSL>IM>LAR. The LAR transcription of EGFR was considerably below the average of the other three subtypes; therefore, targeting EGFR in this subtype may not lead to satisfying treatment outcomes. Similarly, the response of BL1/2 cells to the anti-EGFR therapy may have an opposite effect due to the EGFR paradox described in past studies, where EGF was reported to induce growth arrest or apoptosis in cells with relatively very high EGFR expression.^29^

#### *Failure of anti-EGFR targeting in TNBC*

Although EGFR is a critical oncogenic driver, clinical studies targeting this receptor have largely failed to improve patient outcomes. The limitations of these studies likely stem from multiple factors, including inadequate patient stratification and the heterogeneity of tumor subtypes. Insufficient patient selection in clinical trials that were not specifically restricted to EGFR-expressing TNBC caused low efficacy of EGFR tyrosine kinase inhibitors.^27^ Emerging evidence indicates that EGFR signalling undergoes fundamental alterations during primary tumor invasion, dissemination, and metastasis in breast cancer.^29^ These dynamic changes necessitate an adaptive therapeutic approach tailored to the evolving molecular landscape of the tumor.

One major challenge lies in the selection of appropriate therapies for TNBC subtypes. For instance, low EGFR expression in the LAR subtype may render EGFR-targeted treatments ineffective, underscoring the critical need for precise TNBC subtype classification in clinical practice.^4^ As shown in Figure 4, patients with BL1/2 TNBC are most likely to respond to anti-EGFR therapies, but the single EGFR expression measurement could support a decision while considering EGFR targeting. Furthermore, resistance mechanisms often involve downstream mutations within key signalling pathways such as RAS/RAF/MEK and PI3K/AKT/mTOR, which contribute to therapeutic failure.^31^

Another notable factor is the EGFRvIII mutation, characterized by the deletion of exons 2–7, which renders the receptor incapable of ligand binding. Despite this, EGFRvIII exhibits low-level constitutive signalling, further amplified by impaired receptor internalization and degradation. This aberrant signalling promotes tumor progression while conferring resistance to conventional EGFR inhibitors and monoclonal antibodies. Alternative strategies, such as immunotherapeutic vaccines targeting EGFRvIII-positive cells, have also been proposed to enhance therapeutic efficacy.^32^

Epithelial–mesenchymal transition, which drives cancer metastases, is often associated with a decline in *EGFR* transcription. This results in cell irresponsiveness to EGRF inhibitors, growth inhibition in the presence of EGF, and is known as the EGFR paradox. The reported underlying mechanisms responsible for EGFR resistance to inhibitors include the translocation of EGFR from the membrane to the nucleus, co-expression of other growth-promoting receptors, and their presence on the cell surface. Moreover, cell lines expressing extremely high levels of EGFR respond to EGF with induction of apoptosis and inhibition of cell proliferation.^29^

Beyond EGFR-specific mechanisms, tumor cells may exploit alternative signalling pathways to sustain survival and metastatic progression. Key receptors implicated in this resistance include the fibroblast growth factor receptor (FGFR), anex-elekto receptor tyrosine kinase (AXL), insulin-like growth factor receptor (IGFR), and vascular endothelial growth factor receptor (VEGFR).^33^ For instance, in esophageal cancer, FGFR2 amplification has been linked to resistance against EGFR inhibitors, while in non-small cell lung cancer, c-MET amplification facilitates resistance through reactivation of ErbB3 signalling.^29^ Silencing the gene alone may not lead to cytotoxicity, but could lead to sensitization to chemotherapeutics such as doxorubicin.^34^

These findings highlight the complexity of EGFR-targeted therapy and underscore the necessity for a multifaceted treatment approach that accounts for tumor evolution, molecular heterogeneity, and compensatory resistance mechanisms. Future strategies must integrate precision oncology frameworks to enhance therapeutic efficacy and improve patient outcomes.

#### *Possible EGFR co-partners in TNBC for dual targeting*

**Figure 5:**
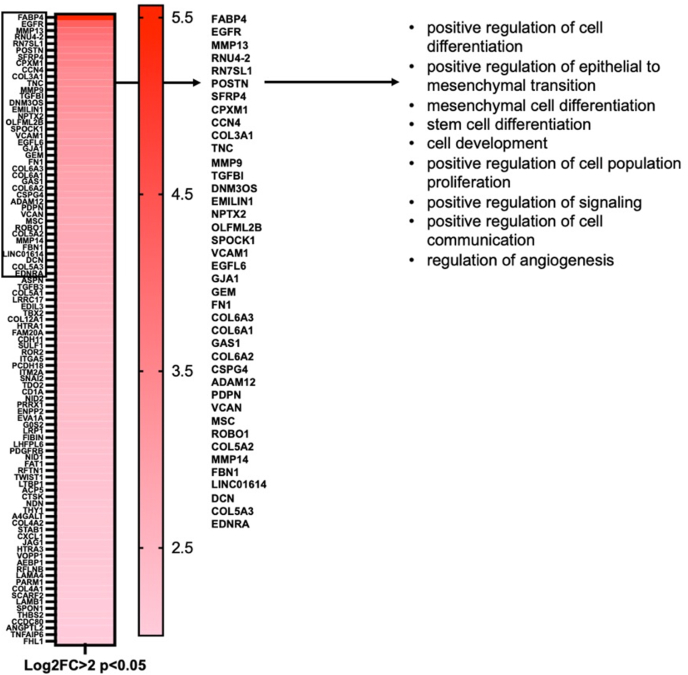
Heatmap presentation of differential EGFR^high^/ESR2^low^/PGR^low^/ERBB2^low^ versus EGFR^low^/ESR2^low^/PGR^low^/ERBB2^low^ gene expression. The genes with the highest differential expression are listed. The analysis covers the processes in which these genes are involved. Results of the analysis were from Xena Browser and PantherGO.

Bearing in mind that inhibition or repression of EGRF activity may not directly cause the collapse of cell functioning or induce the death of cells, but could only make them more vulnerable to some anti-cancer drugs,^34^ we searched for genes overexpressed in parallel to EGFR, serving as co-targets in anti-EGFR approaches. Indeed, the high expression of EGFR in TNBC is associated with high expression of genes, facilitating the functioning of tumor cells. This observation was made while comparing gene expression profiles between TNBCs with EGFR high (EGFR^high^/ESR2^low^/PGR^low^/ERBB2^low^) and low (EGFR^low^/ESR2^low^/PGR^low^/ERBB2^low^) transcription using the TCGA Breast Cancer (BRCA) dataset (Fig. 5). This analysis led to the identification of 96 genes characterized by a 4-fold significant increase in their transcription in EGFR high, versus EGFR low samples. The top 40 genes with the highest overexpression contributed to processes that are crucial for tumor development, growth, and metastases, which include proliferation (*EGFL6*) and signalling (*TNC*), angiogenesis and metastases (*MMP9* and *MMP14*, *VCAM1*), and epithelial-to-mesenchymal transition (*POSTN*). Particular attention should be paid to tumors expressing high EGFR and epithelial-to-mesenchymal transition-relevant genes due to their likely irresponsiveness or reverted responsiveness to anti-EGFR approaches. However, such a distinction, followed by careful functional analysis of cell response to EGF and the gene expression profile, could provide a mechanical explanation for the observed paradox and an adequate specific treatment for these TNBCs.

Below, we list and briefly characterize some examples of proteins functionally involved in cancer initiation, progression, or metastases (which are overexpressed or relatively highly expressed in *EGFR*^*high*^ tumors), as candidates for combination targeting together with anti-EGFR approaches. Their simultaneous inhibition or silencing following EGFR targeting could contribute to a synergistic anti-cancer effect and improve the outcome of single anti-EGFR therapy.

#### FABP4: A lipid metabolism and disease pathogenesis agent

Fatty acid-binding proteins (FABPs) are a family of small, highly conserved cytoplasmic proteins that facilitate the transport of long-chain fatty acids and other hydrophobic ligands within cells.^35^ Among them, adipocyte fatty acid-binding protein (FABP4) is one of the most abundant and has been found to be significantly up-regulated in various malignant solid tumors, correlating to a poor prognosis.^36^ By modulating fatty acid uptake and lipid metabolism, FABP4 plays a crucial role in the metabolic reprogramming of TNBC. Notably, inhibition or genetic ablation of FABP4 has been shown to suppress TNBC tumor progression. Furthermore, the absence of FABP4 has been linked to impaired breast cancer stem cell activity, associated with reduced metastatic potential.^37^ These findings highlight FABP4 as a promising therapeutic target for TNBC treatment.

#### *MMPs and extracellular matrix remodeling in breast cancer*

Matrix metalloproteinases (MMPs) are a family of zinc-dependent endopeptidases that mediate extracellular matrix degradation, facilitating tumor invasion and metastasis. MMPs are expressed by a wide range of cell types, including fibroblasts, keratinocytes, macrophages, endothelial cells, and tumor cells. During tumor progression, the expression of multiple MMP genes is up-regulated, contributing to tumor microenvironment remodeling.^38^

MMP9, a matricellular protein, plays a critical role in extracellular matrix remodeling, modulating cell adhesion molecules, cytokines, and tumor progression. Overexpression of MMP9 has been associated with enhanced cell migration, proliferation, invasion, and apoptosis resistance, serving as a prognostic marker for TNBC patient survival.^39^ Functional studies indicate that MMP9 knockdown significantly impairs TNBC cell growth and invasive capacity. In addition, a combined knockdown of MMP9 and CD151 has been shown to further enhance these inhibitory effects while promoting apoptosis, thus highlighting a potential therapeutic avenue.^40^

MMP14 is a membrane-type MMP that plays an important role in tumor progression and prognosis. The expression of MMP14 is at a much higher level in breast cancer tissue compared with healthy breast tissue. This is associated with tumor cell differentiation, lymph node metastases, and distant metastases.^41^ Therefore, it is a clinically relevant therapeutic target. Its location on the cell surface makes it susceptible to an antibody-mediated blockade. Blocking or silencing MMP14 can limit the progression of cancer and metastasis.^42^

#### VCAM1: A key player in inflammation, cancer, and metastasis

Vascular cell adhesion molecule 1 (VCAM1) gene is a member of the Ig superfamily and encodes a sialoglycoprotein on the cell surface. This 110 kDa glycoprotein is primarily expressed by cytokine-activated endothelial cells during inflammation, though it is also found in macrophages and dendritic cells. As a type I membrane protein, VCAM-1 plays a crucial role in leukocyte and endothelial cell adhesion and signal transduction, and it has been implicated in the development of atherosclerosis and rheumatoid arthritis. Its ability, expressed by endothelial cells in binding tumor cells, suggests a role in facilitating the spread of metastases. Clinically, VCAM-1 expression is correlated with worse outcomes in TNBC and other malignancies, including glioblastoma and ovarian cancer. VCAM1 imaging has been proposed as a diagnostic approach for assessing tumor aggressiveness.^43^

#### CCN4: A critical modulator of tumorigenesis and metastasis

Cellular communication network factor (CCN) proteins are involved in different aspects of tumor development. Despite sharing structural homology, individual CCN proteins exhibit diverse roles across different tumor types and modulate distinct signalling pathways. While some CCN proteins promote tumorigenesis, others contribute to tumor malignancy. These proteins exert their functions through two primary mechanisms: interaction with cell surface receptors and modulation of receptor ligands. Molecular studies have demonstrated that CCN proteins influence key signalling cascades, including ERK, PI3K, and Rho family small GTPases, thereby activating complex signalling cascades within the cellular microenvironment. CCN proteins also regulate major signalling pathways such as integrins, Wnt, and transforming growth factor-β (TGF-β).^44^ These proteins are associated with cell proliferation and cancer risk factors, including chronic inflammation.^45^ CCN4, also known as the WNT1-inducible-signaling pathway protein 1 (WISP-1) (Singh, 2024), exhibits pro-tumorigenicity, particularly in breast cancer. CCN4 mRNA expression is significantly elevated in breast cancer patients, even in the early disease stages, when compared with healthy individuals. CCN4 down-regulates E-cadherin while up-regulating N-cadherin, snail, and β-catenin, facilitating cancer metastasis. Although CCN4 may suppress tumor growth in certain malignancies, it predominantly contributes to cancer progression and metastasis. Notably, CCN4 promotes carcinogenesis by inhibiting N-myc downstream regulated 1 (NDRG), a recognized breast cancer suppressor gene.^45^

#### CDK4/6: Cell cycle regulators and their role in EGFR-driven tumors

Although *CDK4* and *CDK6* do not appear within the top 40 highly expressed genes together with *EGFR*, these two enzymes are crucial for the proliferation of cancer cells and are indispensable for tumor growth. Therefore, their inhibition or silencing may enhance the effect of anti-EGFR therapy.

Cyclin-dependent kinases (CDKs) are key regulators of cell cycle progression. CDK4 and CDK6, members of the CMGC kinase family, function as catalytic subunits within protein kinase complexes that are essential for G1 phase progression and the G1/S transition.^46^ Their activity is tightly regulated by D-type cyclins and CDK inhibitors of the INK4 family. Both CDK4 and CDK6 phosphorylate and regulate the tumor suppressor protein retinoblastoma (Rb), a process frequently dysregulated in various malignancies.^47^ Altered CDK6 expression has been linked to aberrant cell proliferation, impaired cell motility, and oncogenic transformation.^48^

A growing body of evidence suggests that inhibition of the cyclin D/CDK4/6 axis synergizes with EGFR inhibition. While EGFR inhibitors transiently suppress pAKT signalling, tumor cells often maintain AKT activation through a compensatory mechanism involving the death effector domain-containing protein (DEDD). DEDD stabilizes cyclin D1 via its interaction with heat shock recognition protein 70, thereby sustaining a CDK4/6-mediated cell cycle progression. Notably, the cyclin D/CDK4/6-Rb pathway plays a pivotal role in regulating cell cycle checkpoints, a process frequently disrupted in cancer. Selective CDK4/6 inhibitors effectively prevent Rb phosphorylation, inducing G1-phase cell cycle arrest and offering a potential therapeutic strategy.^49^

**Figure 6:**
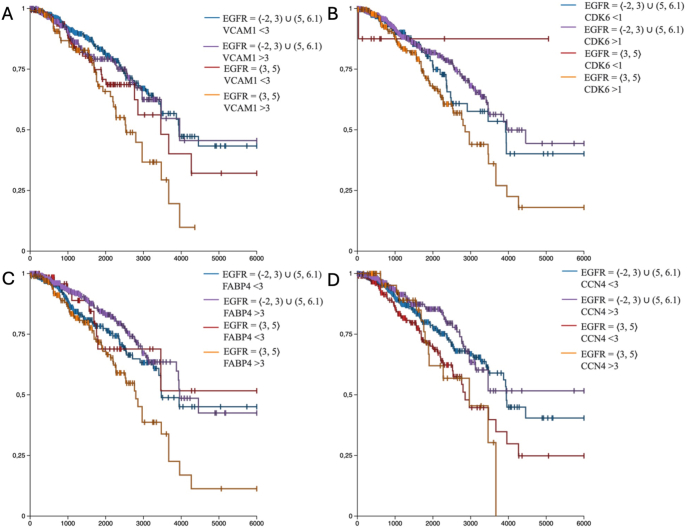
The impact of high EGFR and exemplary four genes on patient overall survival. Kaplan–Meier curves for dual EGFR and VCAM1 (A), CDK6 (B), FABP4 (C), and CCN4 (D) expression based on Xena Browser, GDC TCGA Breast Cancer (BRCA). Values in brackets indicate the range of Log2 normalized values of gene expression, which allows for distinguishing between tumors with simultaneously high EGFR and the other four genes.

Although CDK4/6 dysregulation is not exclusive to TNBC, these kinases are intricately involved in EGFR-dependent signaling cascades. Their expression is strongly correlated with tyrosine kinase activity, suggesting that dual inhibition of CDK4/6 and EGFR may exert a more profound disruption of tumor-promoting pathways.^50^ This underlines the potential of targeting CDK4/6 as part of a broader strategy to overcome EGFR inhibitor resistance in aggressive breast cancers. The effect of high EGFR levels and example genes on the overall survival of patients is shown in Figure 6.

#### *Perspectives for the treatment of EGFR-expressing TNBC with combinatory anti-EGFR strategies*

Since the therapeutic advantage of single inhibition or silencing of EGFR has not always been reported, attempts to target EGFR and other cellular factors or processes, as well as anti-EGFR combination with chemotherapy drugs, have come into focus. Such attempts require delivery techniques and platforms that allow the transportation of two agents into a specific area of the body. So far, researchers have made use of two major types of delivery modes utilizing antibodies and nanocarriers.

#### *Antibody-based EGFR targeting combined with chemotherapy drugs*

Monoclonal antibodies represent the cornerstone of targeted cancer therapy due to their high specificity for tumor-associated antigens. These therapeutic agents act through multiple mechanisms, including direct receptor antagonism, immune-mediated cytotoxicity, and the selective delivery of cytotoxic payloads via antibody-drug conjugates.^51^ EGFR was the first molecular target for monoclonal antibody-based cancer therapy,^52^ making it a prime candidate for nanotechnology-enhanced drug delivery.

In TNBC, high expression of EGFR enables selective drug targeting. Mamot et al^53^ engineered doxorubicin-loaded liposomes with anti-EGFR antibody fragments embedded in pegylated liposomal membranes, thereby improving tumor targeting and drug efficacy. Similarly, Moreira et al^54^ developed DOPE/CHEMS immunoliposomes conjugated with the anti-EGFR antibody cetuximab for the targeted delivery of docetaxel. However, the resistance to anti-EGFR therapies remains a challenge, primarily due to genomic alterations in downstream effectors such as KRAS, NRAS, and PIK3CA, which sustain oncogenic signalling through the RAS–RAF–ERK and PI3K–AKT pathways.^55^

#### *Nanoparticle-based delivery of anti-EGFR factors combined with drugs or other anti-cancer agents*

The quest for effective therapeutic strategies against EGFR has led to the exploration of nanotechnology-based materials and, in particular, nanoparticles, as targeted drug delivery systems. Non-viral vectors, including nanoparticles, play a pivotal role in modern cancer therapeutics by enhancing drug stability, bioavailability, and tumor specificity.^56^ Various nanoparticle-based carriers, such as quantum dots, metal nanoparticles, carbon nanoparticles, dendrimers, and liposomes, have emerged as promising candidates for the efficient delivery of anti-cancer agents.^57^

Dendrimers are highly branched, radially symmetric nanoparticles with a well-defined monodisperse structure composed of a central core, an inner shell, and an outer layer of dendrons.^58^ Their utility in drug delivery arises from two key mechanisms: drug encapsulation via non-covalent interactions and drug conjugation through covalent binding. Both strategies significantly enhance drug solubility, stability and oral bioavailability while enabling the formation of dendriplexes—complexes formed through electrostatic interactions between dendrimers and nucleic acids.^59^ The functional groups at the end of the dendrons determine the nature of the dendrimers. For example, the cationic nature of carbosilane dendrimers is determined by the NMe^3+^ functional groups.^60^

Among nanoparticle-based delivery systems, liposomes remain the most extensively studied for targeted drug transport. These spherical lipid vesicles, typically 50–500 nm in diameter, consist of one or more lipid bilayers that are formed by emulsifying natural or synthetic lipids in an aqueous medium.^61^ Their amphiphilic nature allows encapsulation of both hydrophilic and hydrophobic drugs. A specialized subset of these vesicles, the lipid nanoparticles, feature a homogeneous lipid core and are widely used for the delivery of peptides, small-molecule drugs, and nucleic acids.^62^ Their properties are determined by the selection of phospholipids that form the liposome. To create positively charged liposomes, cationic phospholipids such as 1,2-di-O-octadecenyl-3-trimethylammonium propane, 1,2-dioleoyl-3-trimethylammonium-propane, or N^4^-Cholesteryl-Spermine are often used. The positively charged surface allows the binding of negatively charged molecules such as siRNA due to electrostatic interaction. Hydrophilic therapeutic compounds can be encapsulated within the liposomal core, which is usually filled with water-based solutions, or conjugated to the vesicle’s surface using, *e.g.*, polyethylene glycol (PEG), for targeted delivery.^63^

Nanoparticles offer a versatile platform for the targeted delivery of a wide range of anti-EGFR agents, including antibodies, tyrosine kinase inhibitors (TKIs), and siRNAs. Each of these modalities exploits distinct mechanisms of action, offering unique advantages and limitations.

#### *TKIs and their nanoparticle-based delivery*

Conventional TNBC treatment regimens have often relied on chemotherapeutic agents such as taxanes and anthracyclines, including paclitaxel, docetaxel, doxorubicin, and epirubicin—therapies that have received FDA approval for TNBC management. These are not EGFR-specific agents but are commonly used in anti-TNBC therapy. Among targeted therapies, small-molecule TKIs such as gefitinib and erlotinib have demonstrated efficacy in treating BL2 TNBC,^64^ which are characterized by a high expression of EGFR. Other TKIs like afatinib, brigatinib, cetuximab, osimertinib, dacomitinib, panitumumab, and necitumumab are EGFR-specific inhibitors.^65^ However, systemic use of TKIs is often associated with side effects, including diarrhoea, rash, neutropenia, neuropathy, hepatotoxicity, and hand-foot syndrome.^66^

Nanotechnology-based delivery strategies offer a promising solution for mitigating these adverse effects while also enhancing treatment efficacy. One such approach involves the development of anti-EGFR nanoprotein-liposomes co-loaded with anti-insulin-like growth factor 1 receptor (IGF-1R) kinase inhibitors. The combined targeting of EGFR and IGF-1R has been proposed for the enhancement of therapeutic responses while delaying any resistance mechanism. *In vitro* and *in vivo* experiments demonstrated the simultaneous transport of two inhibitors of EGFR and IGF-1R signalling. The results in cellular models confirmed the toxicity of such a combination, thereby paving the way for further testing in cancer xenograft models. However, such a follow-up study has not been performed as yet.^67^ The use of nanoparticles allows loading of at least two therapeutic agents into one nanoparticle by mixing the encapsulation of the compounds and their binding on the surface. Xue et al described the concept of water-soluble chemotherapeutic encapsulation with siRNA binding to the surface. Here, siRNA was designed to silence the genes responsible for multi-drug resistance, thereby preventing the removal of chemotherapeutics delivered together with siRNA.^68^

#### *siRNA-based double gene silencing with nanocarriers*

Gene silencing through siRNA represents an innovative therapeutic approach for the suppression of oncogenic pathways. siRNA-mediated inhibition of EGFR expression disrupts key processes involved in tumor proliferation, angiogenesis, metastasis, and drug resistance.^69^ RNA interference, the underlying mechanism of siRNA function, involves sequence-specific degradation of target mRNA. The antisense strand of siRNA guides the RNA-induced silencing complex (RISC), which contains the endonuclease Ago2, to cleave mRNA sequences with complementary homology and thereby preventing protein translation.^70^

However, the clinical application of siRNA therapeutics faces several challenges, primarily due to a susceptibility to enzymatic degradation and poor biodistribution. To overcome these obstacles, protective nanocarriers as liposomes or dendrimers, are required to facilitate efficient siRNA delivery. Rationally designed siRNA molecules combined with cationic nanoparticles enhance stability, cellular uptake, and targeted gene silencing.^71^ The cationic character of the liposome surface can be achieved either by selecting the appropriate phospholipid composition or by modifying its surface with ligands capable of binding to siRNA. Ligands such as peptides or proteins can be made from cationic amino acids, while antibodies can be attached to siRNA using a multifunctional peptide linker.^63^ Notably, siRNA-based therapy with liposomes has demonstrated the potential for suppressing EGFR-mediated tumor progression through the precise post-transcriptional regulation of oncogenic gene expression.^72^

The concept of simultaneously silencing multiple genes is emerging as a novel method to improve therapeutic effectiveness. Such a method has already been published as an example of therapy for glioblastoma. Kozielski et al^73^ assumed that the simultaneous transport of siRNAs anti-Robol, *YAP1*, *NKCC1*, and *EGFR* survived using a single carrier like polymeric PBAE nanoparticles. These nanoparticles were characterised by a spherical shape with a diameter of approximately 100 nm and a zeta potential of +18 mV. The use of several siRNAs resulted in enhanced effects, such as the inhibition of the growth and migration of cancer cells. Pillar et al^74^ proposed a different concept based on miRNA-mediated inhibition of ABCE1 and LCP1. *In vivo* experiments confirmed that the double knockdown of Abce1 and Lcp1 reduced tumor aggressiveness and increased survival. Kim et al^75^ studied the use of an anti-EGFR immunoliposome with encapsulated anti-JAK3 siRNA in a single nanocarrier for targeted siRNA delivery to the tumor. The combination of two siRNA types increased the effectiveness of single EGFR targeting. These strategies underscore the potential of nanotechnology and gene silencing to revolutionize cancer treatment based on targeting more than one cancer feature.

## Conclusion

The synergistic inhibition of EGFR and the administration of chemotherapeutic agents enhance the efficacy of anti-cancer treatments.^76^ Therefore, the idea of simultaneous inhibition or silencing of EGFR and other genes overexpressed in TNBC subtypes, which are crucial for cancer well-being, sounds promising, but requires verification in cellular models at first and then, if successful, efficient delivery to the sites of the tumor. Here, nanotechnology offers a robust platform for overcoming the limitations associated with conventional drug delivery methods, providing targeted, efficient, and less toxic therapeutic options. The integration of antibodies, TKIs, and siRNA within nanoparticle-based carriers could hold significant promise for improving any precision approaches in TNBC and other EGFR-driven malignancies. However, this must be preceded by the identification of universal or at least prevailing gene signatures in EGFR-positive cancers for dual targeting and must be followed up by standardized protocols verifying the overexpression in patient biopsies. The copy number duplication, polysomy, mRNA, or protein quantification tests may serve as a starting point for anti-EGFR-based personalized anti-cancer therapy.

## CRediT authorship contribution statement

**Piotr** **Białecki:** Writing – original draft, Visualization, Formal analysis, Conceptualization. **Miłosz Adamiak:** Writing – original draft. **Elżbieta Pędziwiatr-Werbicka:** Writing – review & editing, Supervision. **Agnieszka Robaszkiewicz:** Writing – review & editing, Visualization, Supervision, Formal analysis, Conceptualization.

## Conflict of interests

The authors declared no competing interests.
